# Partial Expression of Genes and Some Proteins Associated with the Dopaminergic Phenotype in Vasopressinergic Neurons of the Supraoptic Nucleus in Intact Mice

**DOI:** 10.3390/ijms27198884

**Published:** 2026-10-06

**Authors:** Tatiana S. Pronina, Dmitry V. Troshev, Liliya K. Dil’mukhametova, Maria O. Shchepina, Michael V. Ugrumov

**Affiliations:** Laboratory of Neural and Neuroendocrine Regulations, Koltzov Institute of Developmental Biology of the Russian Academy of Sciences, Moscow 119334, Russia; tatiana.pronina@mail.ru (T.S.P.); dmitry.vad.troshev@gmail.com (D.V.T.); lili.dilm@gmail.com (L.K.D.); shchepina.maria@yandex.ru (M.O.S.)

**Keywords:** mice, supraoptic nucleus, vasopressinergic neurons, tyrosine hydroxylase, aromatic L-amino acid decarboxylase, vesicular monoamine transporter 2, dopamine transporter, PCR, immunocytochemistry

## Abstract

Nearly forty years ago, it was first discovered that vasopressinergic neurons in the supraoptic nucleus contain tyrosine hydroxylase (TH), the first enzyme of dopamine synthesis. In some mammals, it is expressed only under stress, such as osmotic stimulation and so on (rats); in others it is expressed under normal conditions (tree shrews), and in a third group it is expressed in pathology (humans). This study is devoted to testing the hypothesis that vasopressinergic neurons may express some other proteins of the dopaminergic phenotype in addition to TH. To meet this aim, we assessed the expression of genes encoding proteins of the dopaminergic phenotype, such as TH, aromatic L-amino acid decarboxylase, vesicular monoamine transporter 2, and dopamine transporter, in vasopressin-immunopositive neurons of the supraoptic nucleus of intact C57BL/6 mice isolated by laser microdissection. We demonstrated that vasopressinergic neurons of the supraoptic nucleus express the genes for all of the aforementioned dopaminergic phenotype proteins, with the exception of the dopamine transporter. In a subsequent immunohistochemical study, evidence was obtained that, in addition to TH, some vasopressinergic neurons contain aromatic L-amino acid decarboxylase and, perhaps, vesicular monoamine transporter 2. Further studies are needed to determine whether the dopaminergic phenotype proteins we identified possess specific functional activity.

## 1. Introduction

Several decades have passed since it was first demonstrated that neurosecretory neurons of the hypothalamus, which secrete peptide adenohypophysiotrophic neurohormones into the hypophysial portal circulation, express specific genes and some proteins of the dopaminergic phenotype, typically tyrosine hydroxylase (TH) [1,2,3,4]. TH is the first rate-limiting enzyme of catecholamine synthesis, converting L-tyrosine to L-3,4-dihydroxyphenylalanine [5,6]. In peptidergic neurons of the hypothalamus, with the exception of magnocellular neurons of the supraoptic and paraventricular nuclei, TH is expressed in intact animals [7]. Repeated attempts to determine the functional significance of this enzyme in magnocellular neurons have been unsuccessful [4].

In the supraoptic and paraventricular nuclei of the hypothalamus, TH is expressed in vasopressinergic and oxytocinergic neurons; however, in most mammals, this expression occurs only upon their chronic functional activation [4,8]. The vasopressinergic neurons of the supraoptic nucleus (SON) in rodents are considered the most promising model for studying TH expression, since this nucleus in rodents contains numerous vasopressinergic neurons that greatly outnumber oxytocinergic neurons [2,9,10,11].

The detection of TH expression in vasopressinergic neurons, as one of the proteins of the dopaminergic phenotype [7,12,13], prompted the hypothesis that these neurons may also express other genes and proteins of the dopaminergic phenotype [2,14]. Although previous studies in this area have partially confirmed this hypothesis, the results obtained are quite contradictory and species-specific. For example, in rats, no expression of aromatic L-amino acid decarboxylase (AADC), the second enzyme involved in dopamine synthesis, was detected [2]. In humans, however, PCR detected expression of the gene of AADC in vasopressinergic neurons of the SON; however, this protein could not be detected immunohistochemically [14]. So far, the expression of the AADC gene and synthesis of this enzyme have been only detected in intact shrews [15].

Given that transgenic mice have been widely used in recent decades to study the mechanisms of expression of various functionally significant proteins [16,17,18], we have recently developed promising models of osmotic stimulation of vasopressinergic neurons of the SON in mice [8]. The aim of this study was to evaluate the expression of some genes and synthesis of proteins of the dopaminergic phenotype in vasopressinergic neurons of intact male mice.

## 2. Results

### 2.1. Gene Expression of Some Proteins of the Dopaminergic Phenotype in Vasopressinergic Neurons of the Supraoptic Nucleus in Intact Mice

**Figure 1:**
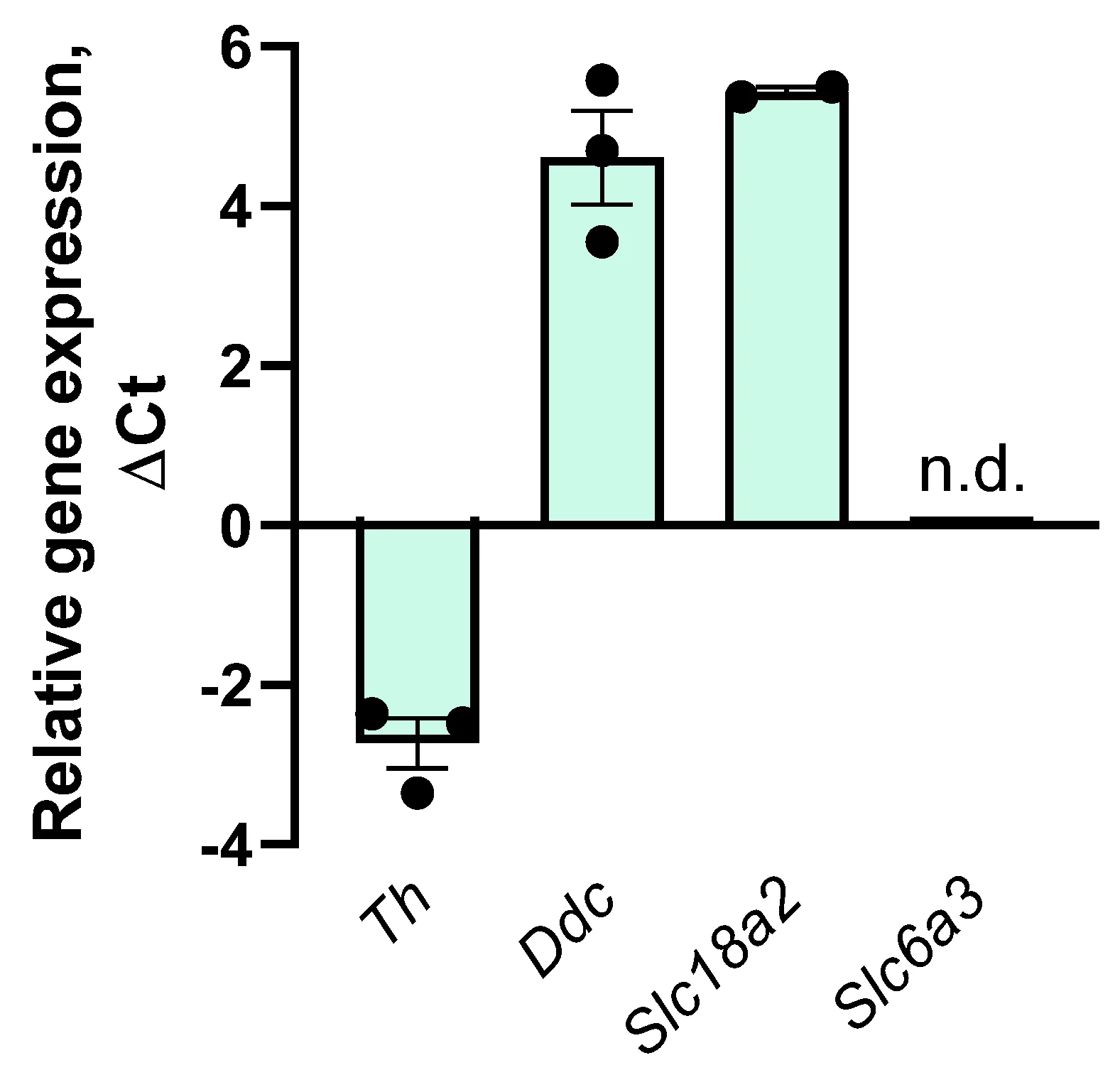
Relative expression of the *Ddc* (aromatic L-amino acid decarboxylase), *Slc6a3* (dopamine transporter), *Slc18a2* (vesicular monoamine transporter 2), and *Th* (tyrosine hydroxylase) genes measured by PCR in vasopressin-immunopositive neurons of the supraoptic nucleus isolated by laser microdissection from brain sections of intact C57BL/6 mice (*n* = 3 probes by 2400 neurons from 6 mice) is presented as ∆Ct (each probe in 3 technical replicates); n.d., not detected. The data are presented as mean with SEM and individual data (dots). Results for Slc18a2 gene expression were presented for two samples, as Slc18a2 mRNA was not detected in the third sample.

In probes of 2400 vasopressinergic neurons prepared using laser microdissection after Carnoy fixation and subsequent immunohistochemistry for vasopressin, TH, AADC and vesicular monoamine transporter 2 (VMAT2), mRNAs were tested. Of the three prepared samples, *Slc18a2* gene expression was detected in two. The *Slc18a2* gene was not detected in the third sample (Figure 1). Only dopamine transporter mRNA was not found (Figure 1). It should be emphasized that upon amplification, TH mRNA reaches the threshold level earlier than AADC mRNA and VMAT2 mRNA. A negative ΔCt value indicates a higher expression level compared to the reference gene (Ct(*Th*) < Ct(*Cyc1*)).

### 2.2. Co-Expression of Some Proteins of the Dopaminergic Phenotype in Vasopressinergic Neurons of the Supraoptic Nucleus in Intact Mice

**Figure 2:**
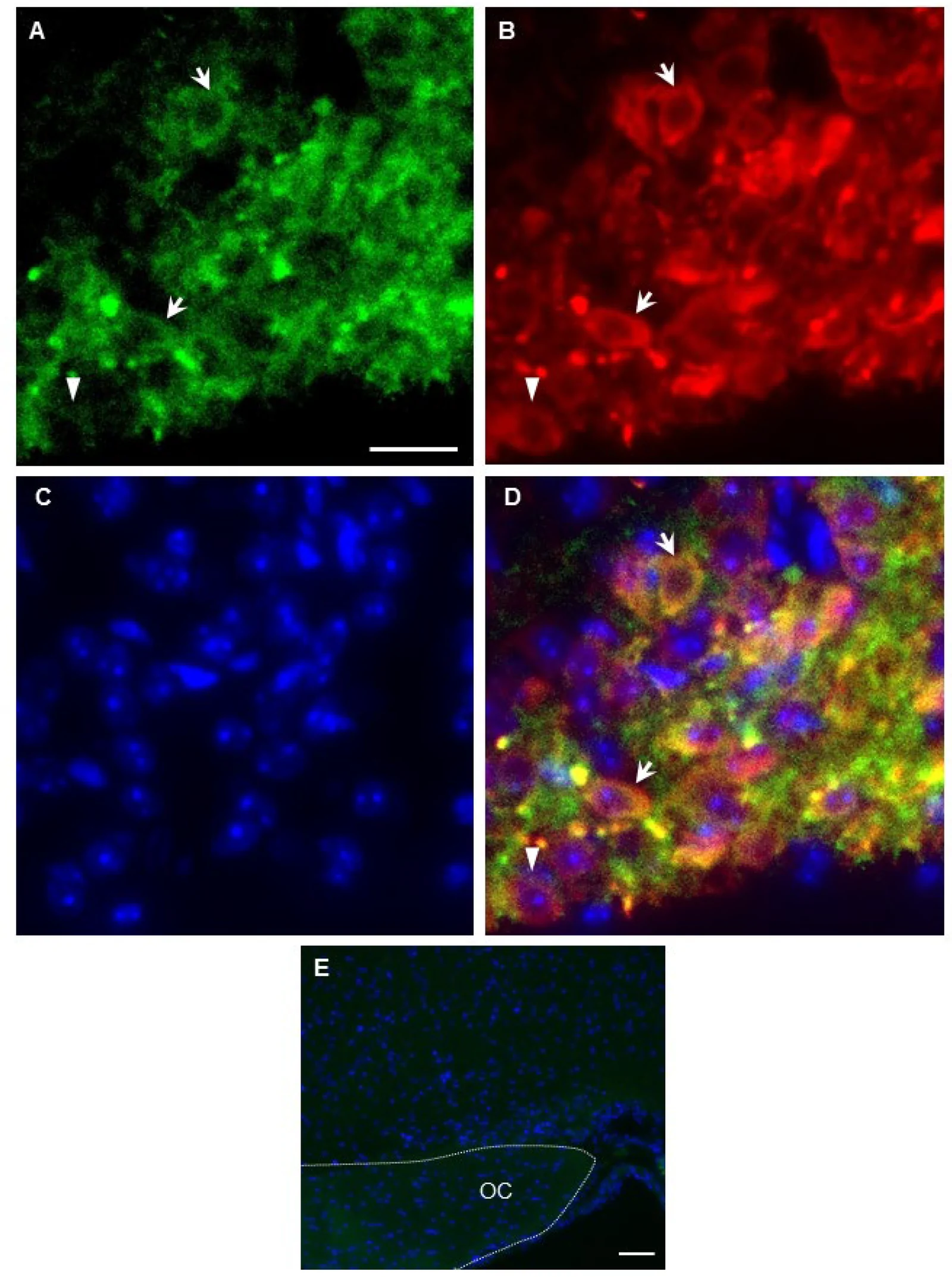
Neurons of the supraoptic nucleus in intact C57BL/6 mice following double immunostaining for aromatic L-amino acid decarboxylase and vasopressin (**A**–**D**) and control—incubation without primary antibodies (**E**). (**A**) Immunostaining for aromatic L-amino acid decarboxylase (green, Alexa 488); (**B**) immunostaining for vasopressin (red, Alexa 555 conjugate); (**C**) neuronal nuclei stained with 4′,6-diamidino-2-phenylindole (blue, DAPI); (**D**) merged (**A**–**C**); (**E**) control incubation without primary antibodies, neuronal nuclei stained with DAPI (blue) and outline optic chiasm (dashed line). Fluorescence microscopy with a 20× objective. Scale bar: (**A**–**D**) 20 µm (see (**A**)), (**E**) 50 µm. Arrowheads, representative neurons immunopositive only for vasopressin; arrows, representative neurons immunopositive for both vasopressin and aromatic L-amino acid decarboxylase.

**Figure 3:**
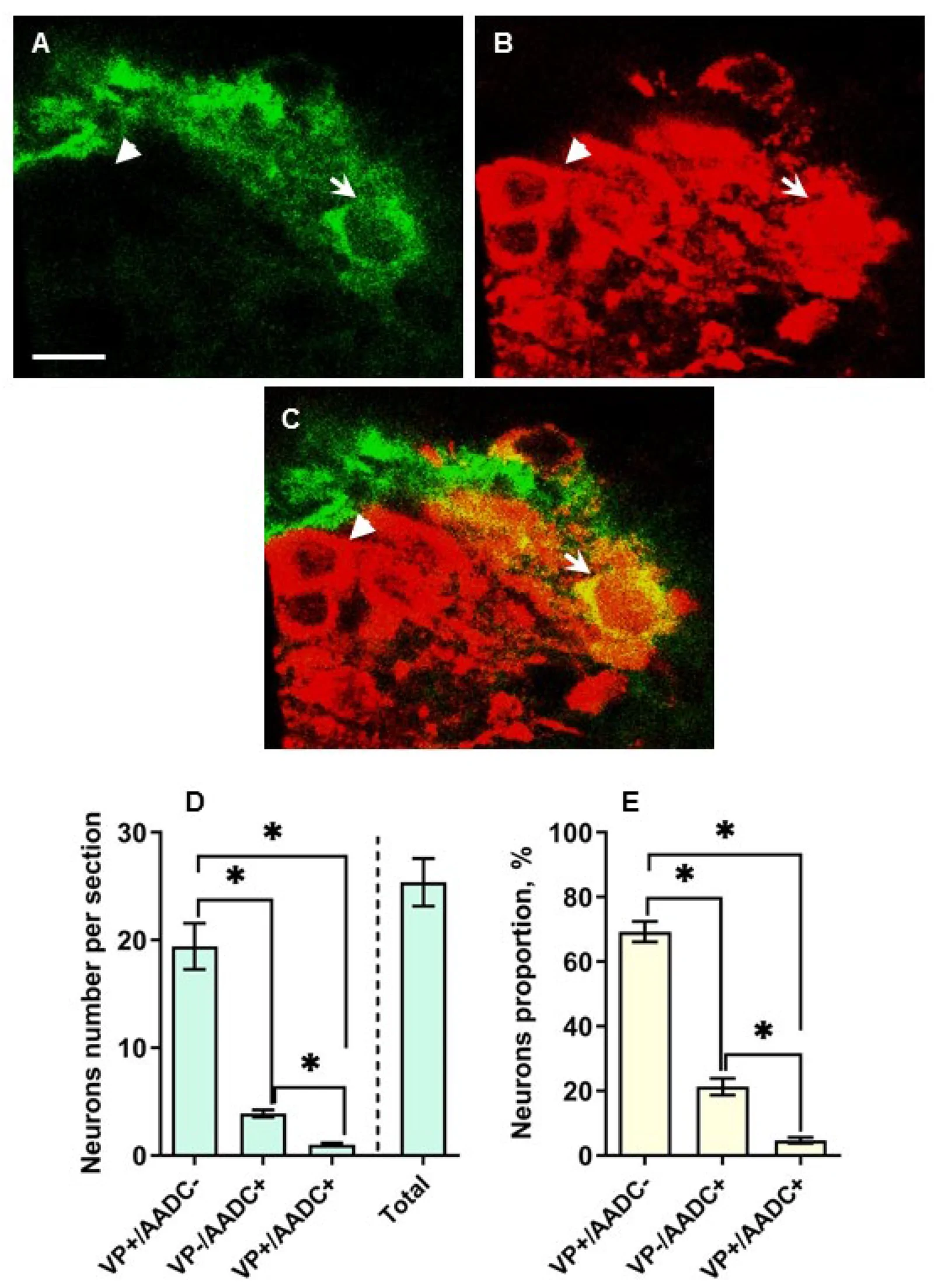
Neurons of the supraoptic nucleus in intact C57BL/6 mice following double immunostaining for aromatic L-amino acid decarboxylase (AADC+) and vasopressin (VP+). (**A**) Immunostaining for aromatic L-amino acid decarboxylase (green, Alexa 488); (**B**) immunostaining for vasopressin (red, Alexa 555 conjugate); (**C**) merged (**A**,**B**); (**D**) neuron number per section; (**E**) the proportion of selected neuronal populations relative to the total number of immunostained neurons, taken as 100%. Confocal microscopy with a 63× objective. Scale bar for (**A**–**C**) 10 μm (see (**A**)). Arrowhead, representative neuron immunostained only for vasopressin (VP+/AADC−); arrow, representative neuron immunostained for vasopressin and aromatic L-amino acid decarboxylase (VP+/AADC+). The data (*n* = 40) at (**D**,**E**) are presented as mean ± SEM. Statistical analysis of the data was performed using the paired *t*-test. * *p* < 0.05, statistically significant difference between the selected groups.

It was shown that vasopressin and AADC colocalize in SON neurons (Figure 2 and Figure 3). According to quantitative analysis of SON neurons double-immunostained for vasopressin and AADC, frontal sections of this nucleus contain, on average, 19.43 ± 2.13 neurons immunostained only for vasopressin; 3.9 ± 0.34 neurons immunostained only for AADC; and 1.03 ± 0.15 neuron immunostained for both vasopressin and AADC (Figure 3D). This means that less than 8% of the vasopressinergic neurons in the SON co-express AADC (Figure 3E).

**Figure 4:**
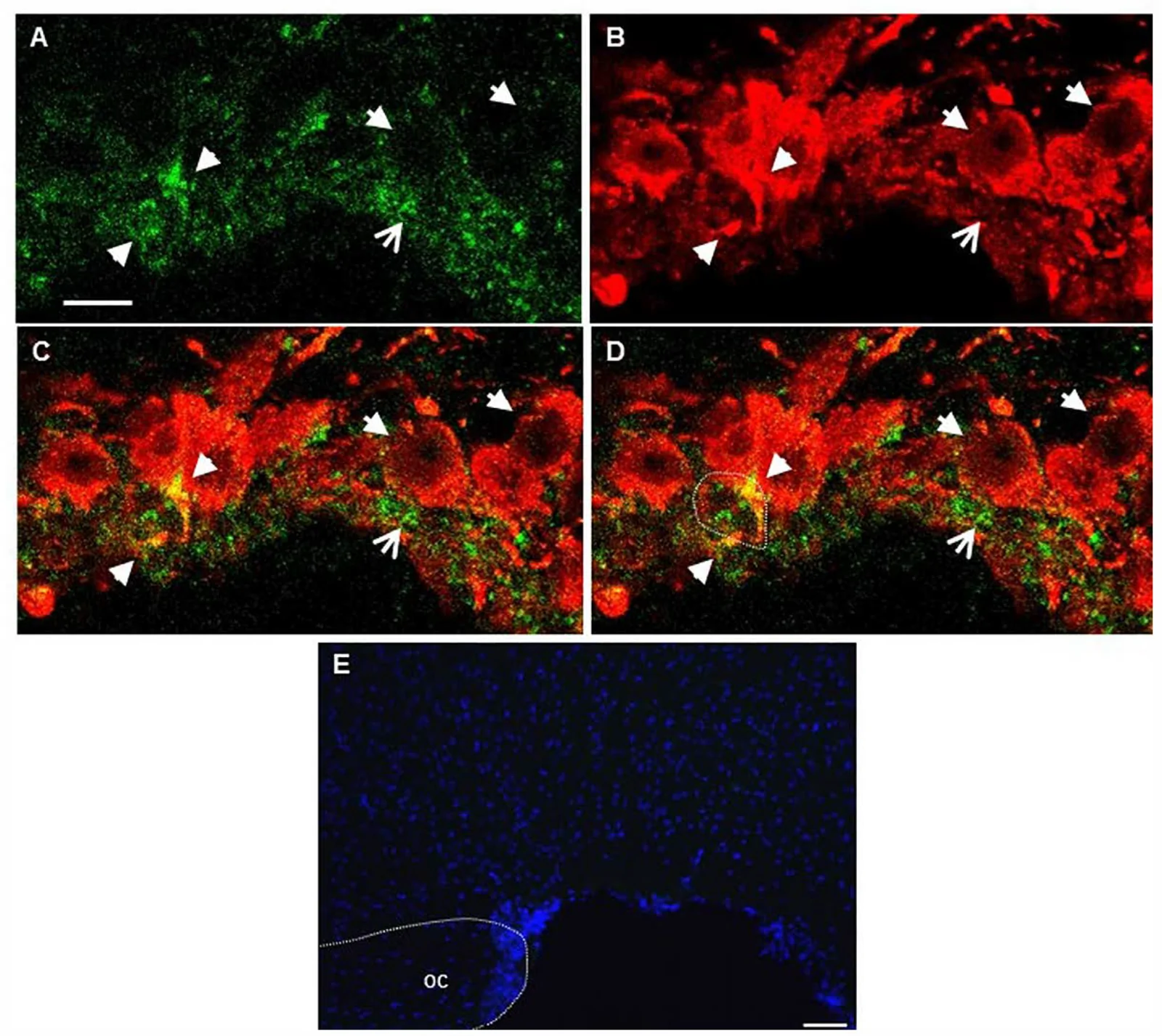
Neurons of the supraoptic nucleus in intact C57BL/6 mice following double immunostaining for vesicular monoamine transporter 2 (VMAT2) and vasopressin. (**A**) Immunostaining for vesicular monoamine transporter 2 (green, Alexa 488); (**B**) immunostaining for vasopressin (red, Alexa 555 conjugate); (**C**) merged (**A**,**B**); (**D**) merge with outlined presumptive double-immunostained neuron (yellow, dashed line, colocalization); (**E**) control incubation without primary antibodies, nuclei stained with 4′,6-diamidino-2-phenylindole (blue, DAPI) and outline optic chiasm (dashed line). Confocal microscopy with a 63× objective (**A**–**D**). Fluorescence microscopy with a 20× objective (**E**). Scale bar for (**A**–**D**) 10 µm (see (**A**)), (**E**) 50 µm. Arrowheads, representative neurons double-immunostained for vasopressin and VMAT2-immunopositive; closed arrows, representative neurons mono-immunostained for vasopressin; open arrows, representative neurons mono-immunostained for VMAT2.

In contrast to vasopressin-immunopositive material and AADC-immunopositive material, which are uniformly distributed in neurons or their fragments, VMAT2-immunopositive material is detected as punctate granular inclusions, which may belong to either neurons or monoaminergic nerve fibers (Figure 4). Consequently, it is difficult to identify VMAT2-containing neurons and impossible to quantify vasopressinergic neurons that contain VMAT2-immunopositive material.

**Figure 5:**
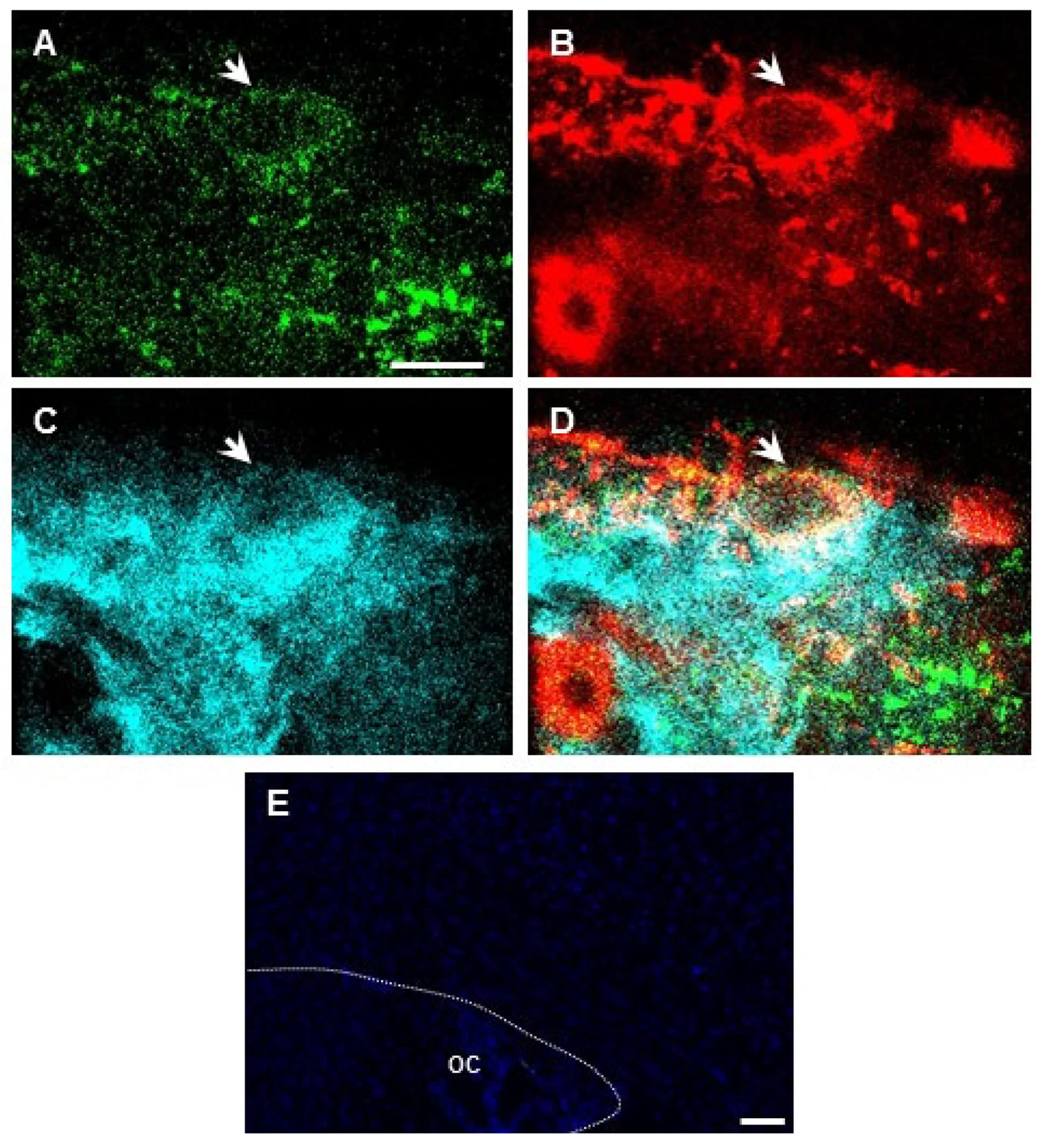
Neurons of the supraoptic nucleus in intact C57BL/6 mice following triple immunostaining for tyrosine hydroxylase, aromatic L-amino acid decarboxylase, and vasopressin. (**A**) Immunostaining for tyrosine hydroxylase (green, Alexa 488); (**B**) immunostaining for vasopressin (red, Alexa 555 conjugate); (**C**) immunostaining for aromatic L-amino acid decarboxylase (blue, Alexa 647); (**D**) merge (**A**–**C**). (**E**) Control incubation without primary antibodies, nuclei stained with 4′,6-diamidino-2-phenylindole (blue, DAPI) and outline optic chiasm (dashed line). Confocal microscopy with a 63× objective (**A**–**D**). Fluorescence microscopy with a 20× objective (**E**). Scale bar for (**A**–**D**) 10 µm (see (**A**)), (**E**) 50 µm. Arrow: representative neuron immunostained for aromatic L-amino acid decarboxylase, tyrosine hydroxylase, and vasopressin.

Following triple immunostaining for TH, AADC, and vasopressin, rare neurons containing all three antigens were detected with a confocal microscope (Figure 5).

**Figure 6:**
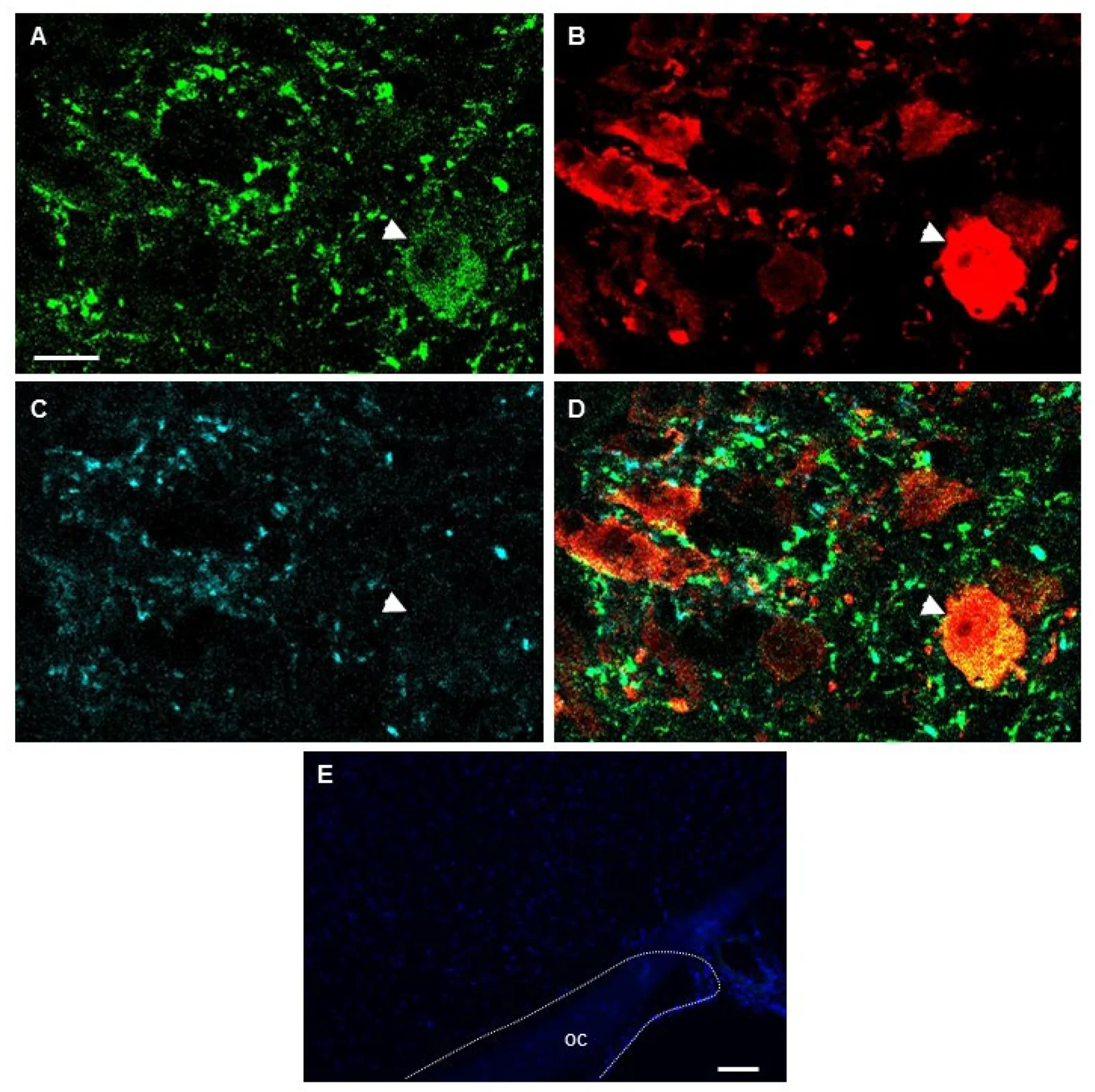
Neurons of the supraoptic nucleus in intact C57BL/6 mice following triple immunostaining for tyrosine hydroxylase, vesicular monoamine transporter 2, and vasopressin. (**A**) Immunostaining for tyrosine hydroxylase (green, Alexa 488); (**B**) immunostaining for vasopressin (red, Alexa 555 conjugate); (**C**) immunostaining for vesicular monoamine transporter 2 (blue, Alexa 647); (**D**) merge (**A**–**C**); (**E**) control incubation without primary antibodies, nuclei stained with 4′,6-diamidino-2-phenylindole (blue, DAPI) and outline optic chiasm (dashed line). Confocal microscopy with a 63× objective (**A**–**D**). Fluorescence microscopy with a 20× objective (**E**). Scale bar for (**A**–**D**) 10 µm (see (**A**)), (**E**) 50 µm. Arrowhead, representative double-immunostained neuron for vasopressin and tyrosine hydroxylase.

After triple immunolabeling for TH, VMAT2, and vasopressin, neurons immunostained for vasopressin and TH were detected by confocal microscopy, but not neurons immunostained for vasopressin and VMAT2 (Figure 6).

**Figure 7:**
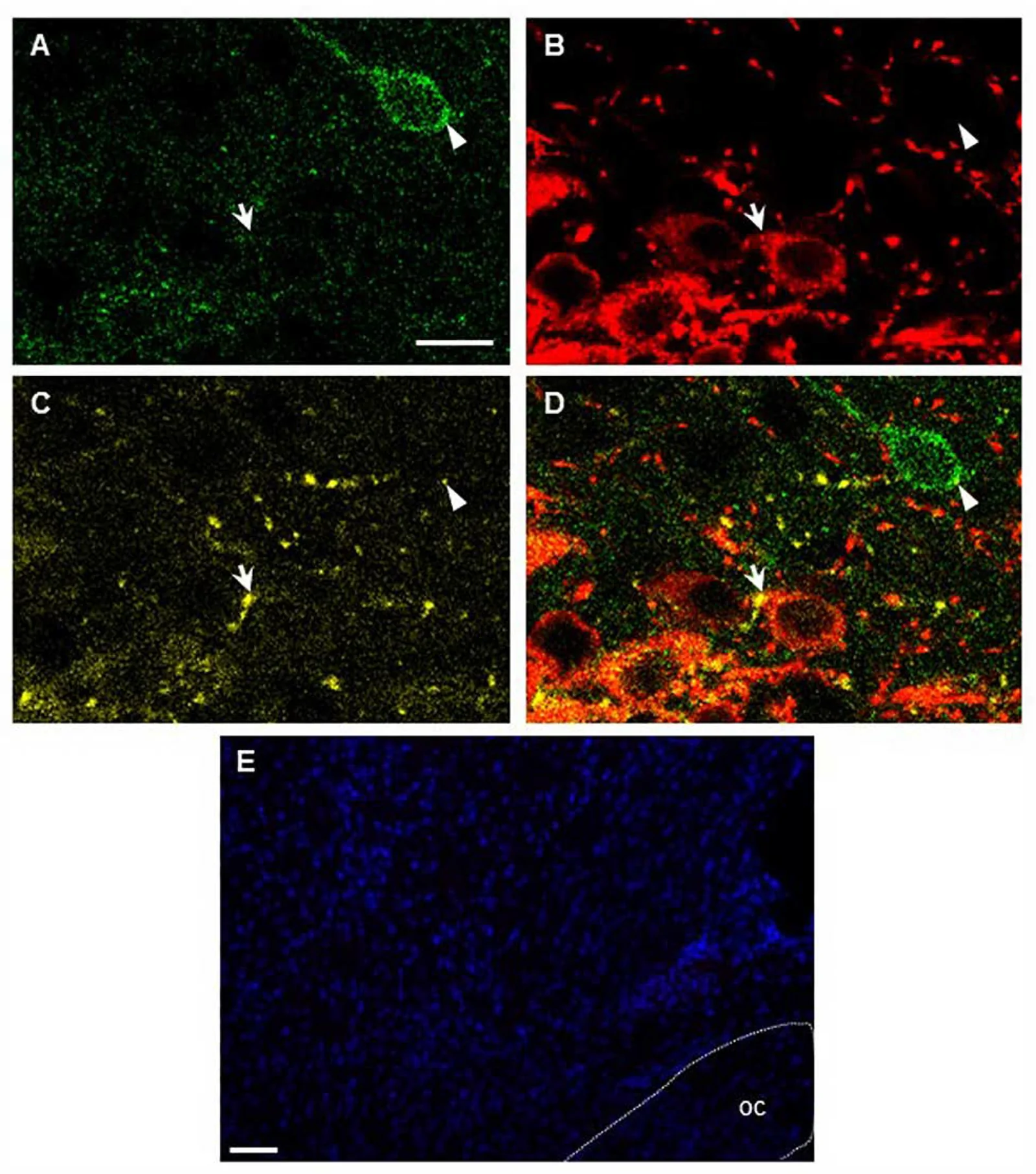
Neurons of the supraoptic nucleus in intact C57BL/6 mice following triple immunostaining for vesicular monoamine transporter 2, aromatic L-amino acid decarboxylase, and vasopressin of neurons. (**A**) Immunostaining for aromatic L-amino acid decarboxylase (green, Alexa 488); (**B**) immunostaining for vasopressin (red, Alexa 555 conjugate); (**C**) immunostaining for vesicular monoamine transporter 2 (yellow, Alexa 647); (**D**) Merge (**A**–**C**); (**E**) control incubation without primary antibodies, nuclei stained with 4′,6-diamidino-2-phenylindole (blue, DAPI) and outline optic chiasm (dashed line). Confocal microscopy with a 63× objective (**A**–**D**). Fluorescence microscopy with a 20× objective (**E**). Scale bar for (**A**–**D**) 10 µm (see (**A**)), (**E**) 50 µm. Arrow, representative vasopressin-immunopositive neuron, possibly containing a punctate inclusion of vesicular monoamine transporter 2-immunopositive material. Arrowhead, representative neuron immunopositive for aromatic L-amino acid decarboxylase and possibly vesicular monoamine transporter 2.

After triple immunolabeling for VMAT2, AADC, and vasopressin, neurons immunostained for vasopressin and VMAT2 were identified using a confocal microscope (Figure 7). However, as noted above, VMAT2-immunopositive material appears as punctate granular inclusions that may belong to either neurons or monoaminergic nerve fibers (Figure 7).

## 3. Discussion

The scientific and technological revolution of recent decades has manifested in the development of advanced methods for studying cells, such as PCR for assessing the expression of protein-coding genes and immunological methods for detecting these proteins using immuno-enzymatic assay in tissues, as well as immunocytochemistry for detecting these proteins in cells [7]. The use of these new methodological approaches in neurobiological research has shown that the brain contains, in addition to dopaminergic and serotonergic neurons, a much larger number of neurons that partially express either the dopaminergic [7,19,20,21] or serotonergic phenotype [7,22]. These neurons often co-express and secrete physiologically active neuropeptides [23].

One population of peptidergic neurons partially expressing the dopaminergic phenotype is represented by vasopressinergic neurons of the magnocellular nuclei of the hypothalamus [24]. About twenty years ago, it was first demonstrated using in situ hybridization and immunocytochemistry on histological sections that these neurons contain TH mRNA and TH-immunopositive protein in addition to vasopressin mRNA and vasopressin-immunopositive material [2]. 

The aim of this study was to assess the expression of some genes and synthesis of proteins of the dopaminergic phenotype in vasopressinergic neurons of intact male mice. It cannot be excluded that the regulation of the expression of not only vasopressin but also some proteins associated with the dopaminergic phenotype in vasopressinergic neurons of the supraoptic nucleus is sex-dependent. However, assessing sex differences in this regulation would require significant additional work, based on determining the phase of the ovulatory cycle in each experiment. This issue could be the subject of future research, especially given that vasopressinergic neurons do not express sex steroid receptors; this type of regulation is mediated by other neurons that express sex steroid receptors and innervate the supraoptic nucleus [25,26].

In the present study, the expression of the TH gene was detected in vasopressin-immunopositive neurons excised from the SON of male rats. However, according to our previous study, this protein lacks enzymatic activity and is unable to convert L-tyrosine to L-3,4-dihydroxyphenylalanine, as occurs in dopaminergic neurons [27,28]. In other words, TH in vasopressinergic neurons resembles TH in dopaminergic neurons only in terms of its immunological characteristics [23].

Based on PCR analysis of vasopressin-immunopositive neurons excised from the supraoptic nucleus of mice, AADC was the second candidate in terms of the expression of genes encoding proteins associated with the dopaminergic phenotype. AADC gene expression was demonstrated earlier in vasopressinergic neurons in humans using the same approach, namely PCR of vasopressinergic neurons isolated by laser microdissection [14,29,30]. However, AADC-immunopositive vasopressinergic neurons were not detected in rats, humans, or monkeys [2,12,31]. Still, a few AADC-immunopositive neurons were detected in the SON of a cat [32], although it remained unclear whether AADC is present in vasopressinergic neurons [33]. 

In this study, we identified AADC-immunopositive vasopressinergic neurons in mice for the first time, indicating the synthesis of this protein. In future research, it is important to determine whether AADC in vasopressinergic neurons has enzymatic activity, unlike TH, which does not. In other words, we will try to test whether AADC is capable of converting L-3,4-dihydroxyphenylalanine into dopamine, as occurs in a number of other hypothalamic neurons that contain only this enzyme [4]. However, it cannot be excluded that AADC in vasopressinergic neurons, like TH, lacks enzymatic activity [23] and shares only immunological similarity with AADC contained in dopaminergic neurons [34].

As demonstrated by PCR, the VMAT2 gene is expressed in vasopressinergic neurons alongside the TH and AADC genes, although to a much lesser extent. In a subsequent immunocytochemical study, we also detected the VMAT2 protein in some vasopressinergic neurons. However, in contrast to vasopressin-immunopositive material and AADC-immunopositive material, which more or less uniformly stain neurons, VMAT2-immunopositive material was observed as punctate granular inclusions, which may belong to either neurons or monoaminergic nerve fibers. If VMAT2 is indeed synthesized in vasopressinergic neurons, it can be assumed that, as in dopaminergic and serotoninergic neurons, it provides the uptake of monoamines from the cytosol into secretory vesicles. Hypothetically, dopamine could be synthesized in vasopressinergic neurons of the supraoptic nucleus (SON) from L-DOPA taken up from the extracellular environment, as it occurs in non-dopaminergic AADC-containing neurons of the arcuate nucleus [35]. However, this is possible only if AADC in SON vasopressinergic neurons is enzymatically active, which is a matter for future research.

We failed to demonstrate the colocalization of AADC and VMAT2 in vasopressinergic neurons, which is in line with previous studies. Indeed, previous attempts to detect the expression of these proteins in SON neurons in other mammals are inconsistent. Thus, AADC was identified in SON neurons in monkeys, cats, and shrews [15,31,32], whereas VMAT2 was detected only in shrews [15]. In humans, the AADC gene is expressed [14], yet the AADC protein itself has not been detected [12].

When testing for the expression of genes encoding selected proteins of the dopaminergic phenotype in excised vasopressinergic neurons, the only mRNA that we failed to detect was the dopamine transporter mRNA. The dopamine transporter had also not been previously detected in neurons of the magnocellular nuclei in rats [36]. The absence of the dopamine transporter in neurons expressing dopamine-synthesizing enzymes [36] is not a unique feature of vasopressinergic neurons. Indeed, such neurons have also been found in other regions of the hypothalamus, primarily in the arcuate nucleus [5,28].

## 4. Materials and Methods

### 4.1. Animals and Experimental Procedures

**Figure 8:**
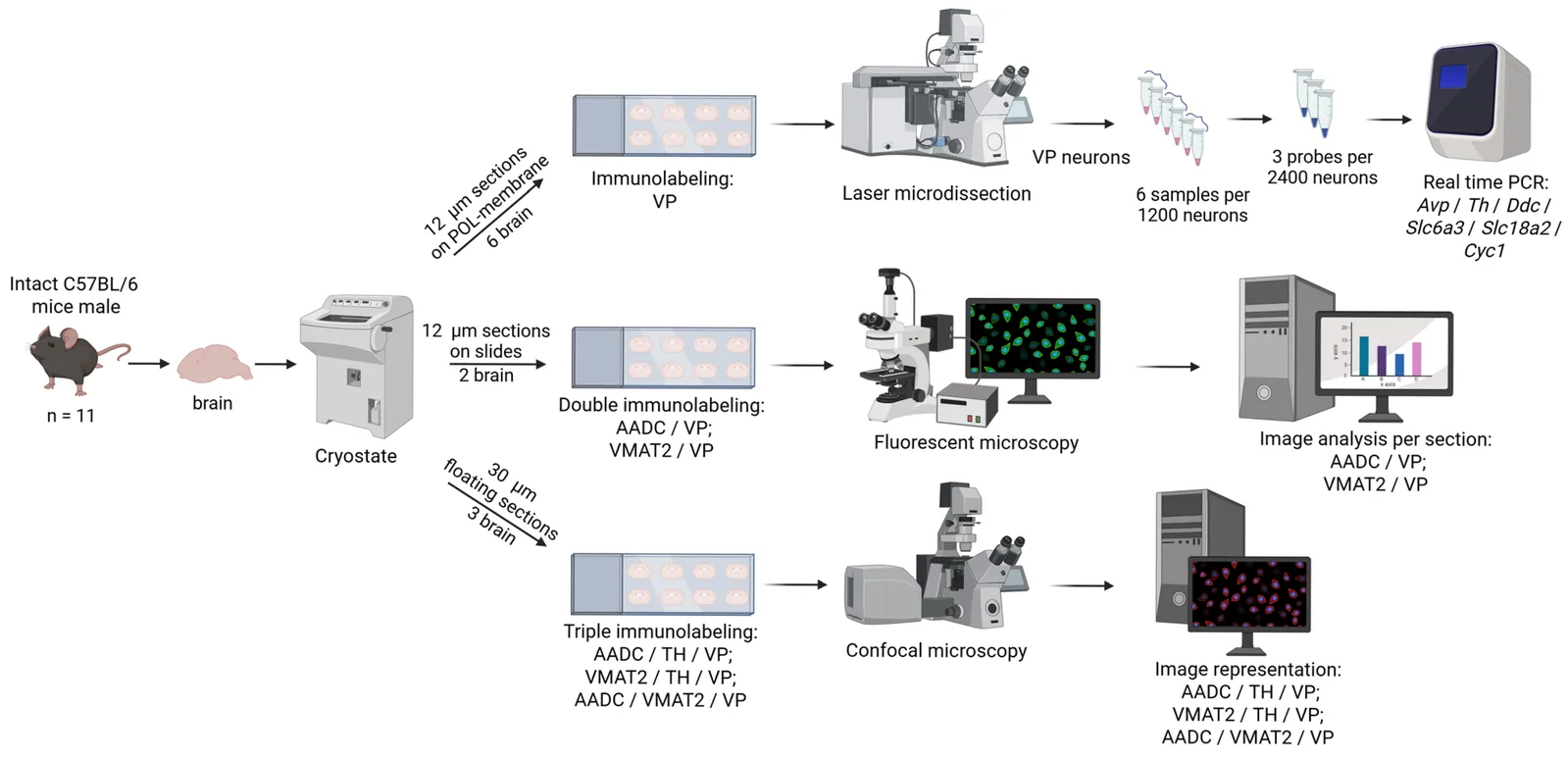
Schematic representation of the methodology used to study the supraoptic nucleus of intact C57BL/6 mice: real-time polymerase chain reaction (PCR) after laser micro-dissection of vasopressin-immunopositive neurons (top row) and double immunolabeling of vasopressin and some proteins of dopaminergic phenotype on histological sections (bottom row). AADC, aromatic L-amino acid decarboxylase; POL-membrane, 0.9-μm polyester membrane; VMAT2, vesicular monoamine transporter 2; VP, vasopressin; *Avp*, the vasopressin gene; *Ddc*, the aromatic L-amino acid decarboxylase gene; *Slc6a3*, the dopamine transporter gene; *Slc18a2*, the vesicular monoamine transporter 2 gene; *Th*, the tyrosine hydroxylase gene. Created in BioRender. Blokhin, V. (2026) https://BioRender.com/27f4rju.

C57BL/6 mice were obtained from the “Stolbovaya” breeding facility (SCBMT RAMS, Stolbovaya, Moscow, Russia). Male mice (*n* = 11) aged 8–10 weeks and weighing 22–25 g were used in the study (Figure 8). The animals were housed at a temperature of 22 ± 1 °C, on a 12 h day/night cycle, and had free access to food and water.

#### 4.1.1. Preparation of Brain for Laser Microdissection and Further Gene Detection

Intact male mice (*n* = 6) were anesthetized with 2.4% isoflurane (Laboratorios Karizoo, Barselona Spain) using a SomnoSuite anesthetic machine (Kent Scientific, Torrington, CT, USA). The mice were then decapitated, and the brains were removed from the skulls and frozen in liquid nitrogen vapor according to a previously published protocol [37]. The resulting material was stored at −70 °C.

#### 4.1.2. Preparation of Brains for Immunohistochemical Analysis of Neurons

Intact mice (*n* = 5) were anesthetized with 2.4% isoflurane using an anesthetic machine. The mice were then decapitated, and the brains were removed from the skulls and fixed in 4% paraformaldehyde in 0.1 M phosphate buffer (pH 7.2–7.4) at +4 °C for 12 h, then washed three times in 0.02 M phosphate-buffered saline (PBS, pH 7.2–7.4), with each wash lasting 30 min. The brains were then incubated in 20% sucrose in 0.02 M PBS at 4 °C for 24 h, frozen in hexane at −40 °C, and stored at −70 °C until immunohistochemistry was performed.

### 4.2. Laser Microdissection of Neurons Immunostained for Vasopressin

Polyester membranes measuring 0.9 μm (Frame Slides, Leica, Wetzlar, Germany) were treated with RNAse Clean (BioinnLabs, Rostov-on-Don, Russia) for 5 min, rinsed twice with diethylpyrocarbonate-treated water (Evrogen, Moscow, Russia) for 30 s each time, then rinsed twice with 96° alcohol (Russia) and dried in a laminar flow hood under UV light for 45 min. The membranes were then stored in a closed, dry chamber at 20 °C until section mounting, for no longer than 40 min. The cryostat (Leica CM1950, Leica, Wetzlar, Germany) was cleaned inside and outside, and all instruments within the cryostat were treated with 6% hydrogen peroxide and then with 96° alcohol. Afterward, the cryostat’s inner chamber was irradiated with UV light for 2.5 h.

In the cryostat at a holder and chamber temperature of −17 °C, 12-μm-thick serial frontal brain sections were prepared at the level of SON over the distance from −0.46 mm to −1.06 mm in the rostro-caudal direction relative to Bregma in accordance with the mouse brain atlas [38]. The sections were mounted on the membranes and fixed in Carnoy’s solution containing 60% 96° ethanol, 30% chloroform (Sigma-Aldrich, St. Louis, MO, USA) and 10% glacial acetic acid (Sigma-Aldrich, St. Louis, MO, USA) for 3 min at −20 °C.

After fixation, the sections were rinsed sequentially as follows: twice for 30 s in 96° ethanol, twice for 30 s in 70° ethanol, and twice for 1 min in 0.02 M PBS containing 1 U/μL RNase inhibitor (SUPERase-in, Invitrogen, Thermo Fisher Scientific, Waltham, MA, USA). They were then incubated with rabbit anti-vasopressin antibodies conjugated to Alexa Fluor 555 (Abcam, Cambridge, UK) (1:200) in 0.02 M PBS containing 1 U/μL of the RNase inhibitor SUPERase-in for 75 min at 20 °C. The sections were then washed three times for 1 min each in PBS containing 1 U/μL of the RNase inhibitor SUPERase-in, followed by two washes for 30 s each with 96° alcohol, and were dried for 15 min. All solutions were prepared using diethylpyrocarbonate-treated water.

Laser microdissection of vasopressin-immunopositive neurons of SON was performed on a microdissector (Leica 7000, Wetzlar, Germany) at 40× magnification, laser power 35, laser aperture 7, speed 20, and pulse frequency 1045 (The Core Centrum of the Koltzov Institute of Developmental Biology RAS). The isolated neurons were collected in the caps of 0.6 mL tubes filled with 50 μL of TRI Reagent (Sigma-Aldrich, St. Louis, MO, USA). Six samples from 6 male mice were collected, each containing 1200 vasopressin-immunopositive neurons. The collected samples were frozen in liquid nitrogen and stored at −70 °C.

### 4.3. RNA Extraction and Real-Time PCR

Six initial samples—each containing 1200 vasopressin-immunopositive neurons isolated via laser microdissection from 6 mice—were pooled in pairs to form three final probes of 2400 neurons each for PCR analysis. The probe volumes were adjusted to 1 mL each using TRI Reagent. The volume of TRI Reagent in probes containing brain tissue, used as a control to assess mRNA integrity, was also adjusted to 1 mL. Next, 100 μL of 1-bromo-3-chloropropane (Sigma-Aldrich, St. Louis, MO, USA) was added to each probe, and this solution was incubated for 15 min at 20 °C with regular mixing on a vortex. The aqueous and organic phases were separated by centrifugation of the solution for 15 min at 21,000× *g* and 4 °C.

The aqueous phase containing RNA was transferred to a new tube, to which 500 μL of isopropyl alcohol (Sigma-Aldrich, St. Louis, MO, USA) and 2 μL of glycogen (Thermo Fisher Scientific, Waltham, MA, USA) were added to enhance RNA precipitation. The solution was vortexed and incubated for 10 min at 20 °C, after which the RNA was precipitated by centrifugation for 20 min at 21,000× *g* and 4 °C. The supernatant was removed, and the precipitate was washed three times with 1 mL of 80% ethanol and centrifuged for 10 min at 21,000× *g* and 4 °C. After the final centrifugation, the ethanol was removed and the RNA precipitate was air-dried for 15 min and then dissolved in 11 μL of RNase-free and DNase-free water. The RNA concentration in all probes was measured using a NanoDrop 8000 spectrophotometer (Thermo Fisher Scientific, Waltham, MA, USA).

RNA integrity was further assessed by electrophoresis in a 1% agarose gel containing ethidium bromide (Thermo Fisher Scientific, Waltham, MA, USA). Residual genomic DNA was removed using DNase I, RNase-free (Thermo Fisher Scientific, Waltham, MA, USA), according to the manufacturer’s protocol. Complementary DNA was synthesized from 15 ng of total RNA using the Maxima H Minus First Strand cDNA Synthesis Kit (Thermo Fisher Scientific, Waltham, MA, USA) according to the manufacturer’s instructions. qPCR was performed using qPCRmix-HS SYBR+LowROX (Evrogen, Moscow, Russia) on a QuantStudio 12k Flex thermal cycler (Applied Biosystems, Thermo Fisher Scientific, Waltham, MA, USA). Oligonucleotide primers (Table 1, Evrogen, Moscow, Russia) were designed within the coding sequence (CDS) of the target genes and were used to determine the expression of the following genes: *Th*, *Ddc*, *Slc6a3*, *Slc18a2*, and *Cyc1*. One microgram of cDNA was used for qPCR. The expression of *Th*, *Ddc*, *Slc6a3*, and *Slc18a2* was calculated using the ΔCt method. *Cyc1* was taken as a housekeeping gene.

∆Ct = (Ct(*target gene*) − Ct(*Cyc1*),

where Ct is the cycle threshold value corresponding to the point at which the amplification curve intersects the fixed threshold line.

Validation of primer pairs used for PCR was performed (Figure S1) and the quality of the effective removal of genomic DNA from the probes (Figure S2) was tested.

### 4.4. Immunohistochemistry

Using a cryostat set at a holder temperature of −21 °C and a chamber temperature of −20 °C (Leica CM1950, Leica, Wetzlar, Germany), 12 μm thick serial frontal brain sections of 2 mice and 30 μm thick serial frontal brain sections of 3 mice were cut at the levels of SON over the distance from −0.46 mm to −1.06 mm in the rostro-caudal direction relative to Bregma in accordance with the mouse brain atlas [38]. Every second 12 μm thick section was mounted on glass slides and every third 30 μm thick section was placed in PBS and incubated as a free-floating section.

For double immunolabeling of vasopressin and AADC 12 μm thick sections were sequentially incubated in PBS containing (i) 3% bovine serum albumin (Sigma-Aldrich, St. Louis, MO, USA) and 0.3% Triton X-100 (Sigma-Aldrich, St. Louis, MO, USA) for 1 h at 20 °C; (ii) rabbit antibodies against AADC (1:500, Synaptic Systems GmbH, Goettingen, Germany), 1% bovine serum albumin, and 0.1% Triton X-100, for 20 h at 20 °C; (iii) goat anti-rabbit gamma globulin antibodies conjugated with Alexa-Fluor-488 (1:1000, Invitrogen, Thermo Fisher Scientific, Waltham, MA, USA, A11015), for 2 h at 20 °C; and (iv) Alexa-Fluor-555-conjugated rabbit anti-vasopressin antibodies (1:1500, Abcam, Cambridge, UK), for 20 h at 20 °C. Other 12 μm thick sections used for double immunostaining for vasopressin and VMAT2 were sequentially incubated in PBS containing (i) 3% bovine serum albumin and 0.3% Triton X-100, for 1 h at 20 °C; (ii) rabbit anti-VMAT2 antibody (1:300, Millipore, Burlington, MA, USA), 1% bovine serum albumin, and 0.1% Triton X-100, for 20 h at 20 °C; and (iii) Alexa-Fluor-488-conjugated goat anti-rabbit gamma globulin antibodies (1:1000, Invitrogen, Thermo Fisher Scientific, Waltham, MA, USA, A32733), for 2 h at 20 °C, and (iv) Alexa-Fluor-555-conjugated rabbit anti-vasopressin antibodies (1:1500, Abcam, Cambridge, UK), for 20 h at 20 °C. After each incubation, the sections were washed in PBS. After the last wash, the sections were embedded in a medium containing the nuclear stain DAPI (Abcam, Cambridge, UK).

For triple immunolabeling of vasopressin, AADC, and VMAT2, 30 μm thick free-floating sections were sequentially incubated in PBS containing (i) 3% bovine serum albumin, 0.3% Triton X-100, and 3.5% Blocking reagent (M.O.M. Immunodetection kit, Vector Laboratories, Newark, CA, USA), for 1 h at 20 °C; (ii) 3% bovine serum albumin, 0.3% Triton X-100, and 8% Protein concentrate (M.O.M. Immunodetection kit, Vector Laboratories, Newark, CA, USA), for 20 min at 20 °C; (iii) mouse antibodies against AADC (1:300, Synaptic Systems, Germany), 1% bovine serum albumin, 0.1% Triton X-100, and 8% Protein concentrate for 20 h at 4 °C; (iv) goat anti-mouse gamma globulin antibodies conjugated with Alexa-Fluor-488 (1:1000, Invitrogen, Thermo Fisher Scientific, Waltham, MA, USA), for 2 h at 20 °C; (v) rabbit anti-VMAT2 antibody (1:300, Millipore, Burlington, MA, USA), 1% bovine serum albumin, and 0.1% Triton X-100, for 20 h at 20 °C; and (vi) Alexa-Fluor-647-conjugated goat anti-rabbit gamma globulin antibodies (1:1000, Invitrogen, Thermo Fisher Scientific, Waltham, MA, USA), for 2 h at 20 °C; and (vii) Alexa-Fluor-555-conjugated rabbit anti-vasopressin antibodies (1:1800, Abcam, Cambridge, UK), for 20 h at 20 °C. After each incubation, the sections were washed in PBS. The sections were then embedded in a medium containing the nuclear stain DAPI (Abcam, Cambridge, UK).

For triple immunolabeling of vasopressin, AADC, and TH, 30 μm thick free-floating sections were sequentially incubated in PBS containing (i) 3% bovine serum albumin, 0.3% Triton X-100, and 3.5% Blocking reagent (M.O.M. Immunodetection kit), for 1 h at 20 °C; (ii) 3% bovine serum albumin, 0.3% Triton X-100, and 8% Protein concentrate (M.O.M. Immunodetection kit), for 20 min at 20 °C; (iii) mouse antibodies against AADC (1:300, Synaptic Systems, Germany), 1% bovine serum albumin, and 0.1% Triton X-100, and 8% Protein concentrate for 20 h at 4 °C; (iv) donkey anti-mouse gamma globulin antibodies conjugated with Alexa-Fluor-647 (1:1000, Invitrogen, Thermo Fisher Scientific, Waltham, MA, USA), for 2 h at 20 °C; (v) sheep polyclonal antibodies to TH (1:700) (Millipore, Burlington, MA, USA, AB 1542), 1% bovine serum albumin, and 0.1% Triton X-100, for 20 h at 20 °C; (vi) Alexa-Fluor-488 donkey antibodies against sheep gamma globulins (1:1000, Invitrogen, Thermo Fisher Scientific, Waltham, MA, USA, A11015), for 2 h at 20 °C; and (vii) Alexa-Fluor-555-conjugated rabbit anti-vasopressin antibodies (1:1800, Abcam, Cambridge, UK), for 20 h at 20 °C. After each incubation, the sections were washed in PBS. The sections were then embedded in a medium containing the nuclear stain DAPI (Abcam).

For triple immunolabeling of vasopressin, VMAT2, and TH, 30 μm thick free-floating sections were sequentially incubated in PBS containing (i) 3% bovine serum albumin, and 0.3% Triton X-100 for 1 h at 20 °C; (ii) rabbit anti-VMAT2 antibody (1:300, Millipore, Burlington, MA, USA), 1% bovine serum albumin, and 0.1% Triton X-100, for 20 h at 20 °C; (iii) Alexa-Fluor-647-conjugated goat anti-rabbit gamma globulin antibodies (1:1000, Invitrogen, Thermo Fisher Scientific, Waltham, MA, USA), for 2 h at 20 °C; (iv) sheep polyclonal antibodies to TH (1:700) (Millipore, Burlington, MA, USA, AB 1542), 1% bovine serum albumin, and 0.1% Triton X-100, for 20 h at 20 °C; (v) Alexa-Fluor-488 donkey antibodies against sheep gamma globulins (1:1000) (Invitrogen, Thermo Fisher Scientific, Waltham, MA, USA, A11015), for 2 h at 20 °C; and (vi) Alexa-Fluor-555-conjugated rabbit anti-vasopressin antibodies (1:1800, Abcam, Cambridge, UK), for 20 h at 20 °C. After each incubation, the sections were washed in PBS. The sections were then embedded in a medium containing the nuclear stain DAPI (Abcam, Cambridge, UK).

For all immunohistochemical reactions, controls were performed by incubating sections without primary antibodies (Figure 2, Figure 4, Figure 5, Figure 6 and Figure 7). Additionally, immunostaining for AADC and VMAT2 in dopaminergic neurons of the substantia nigra was used as a positive control (Figure S3).

### 4.5. Microscopy and Image Analysis

The SON neurons double-immunostained for vasopressin and for either AADC or VMAT2 were examined under a conventional fluorescence microscope (Leica DMi8 M, Leica Camera AG, Wetzlar, Germany) (The Core Centrum of the Koltzov Institute of Developmental Biology RAS) using the objective 20× (HC PL FLUOTAR L 20×/0.40 DRY) and analyzed by LAS X (Leica Application Suite X 3.7.6.25997, Leica Microsystems CMS GmbH, Wetzlar, Germany).

The SON neurons triple-immunostained for vasopressin, AADC, and VMAT2 or vasopressin, AADC, and TH or vasopressin, TH, and VMAT2 were examined under confocal microscope Leica SP5 (Leica, Wetzlar, Germany) (The Core Centrum of the Koltzov Institute of Developmental Biology RAS) using the immersion objective 63× (HCX PL APO CS 63.0 × 1.40 OIL UV), and controlled by LAS AF software (Leica Application Suite X 3.7.6.25997, Leica Microsystems CMS GmbH, Wetzlar, Germany). Each fluorophore was assigned to a separate acquisition track. Fluorescence of vasopressin was excited by a laser with a wavelength of 561 nm, fluorescence of VMAT2 was excited by a laser with a wavelength of 633 nm, fluorescence of TH was excited by a laser with a wavelength of 488 nm, and fluorescence of AADC was excited by a laser with a wavelength of 488 nm or 633 nm, depending on the type of triple immunolabeling. The Z-stack was performed with optical sections of 2 µm size. Image processing was done with LAS X software (Leica Application Suite X 3.7.6.25997, Leica Microsystems CMS GmbH, Wetzlar, Germany).

### 4.6. Statistics

The number of neurons (labeled with AADC alone, vasopressin alone, or the dual marker for AADC and vasopressin) was determined in 40 sections of the supraoptic nucleus from intact C57BL/6 mice following dual immunostaining for AADC and vasopressin. The proportion of the identified neuronal populations (labeled with AADC alone, vasopressin alone, or the dual marker for AADC and vasopressin) was calculated relative to the total number of immunostained neurons, which was set at 100%. Statistical analysis was performed using the GraphPad Prism 9 software (GraphPad Software, San Diego, CA, USA). For pairwise comparisons, the paired *t*-test was used. The results are presented as mean ± SEM. Differences were considered significant at *p* ≤ 0.05.

## 5. Conclusions

Thus, the present study demonstrates that vasopressinergic neurons of the magnocellular nuclei of the hypothalamus express some dopaminergic phenotype proteins such as AADC and VMAT2.

## Figures and Tables

**Table 1:** Oligonucleotide primers used for RT-PCR.

Gene	Forward Primer	Reverse Primer
*Th*	TCAGAGGAGCCCGAGGTC	GGGCGCTGGATACGAGAG
*Ddc*	TCCCCACGGCTAGCTCATACCC	TTCCCCAGCCAGTCCATCATCA
*Slc6a3*	CATCAACCCACCGCAGAC	GAAGGCACCTCCACCATTT
*Slc18a2*	ATTGGCTTTCCTTGGCTCAT	GGTACGGCTGGACATTATTCTG
*Cyc1*	GCGGCCAGGGAAGTTGT	GCCAGTGAGCAGGGAAAATAC

## Data Availability

The data presented in this study are available on request from the corresponding author. The data are not publicly available due to legal issues.

## Supplementary Materials

The following supporting information can be downloaded at https://www.mdpi.com/article/10.3390/ijms27198884/s1.

## Abbreviations

The following abbreviations are used in this manuscript:

AADC	aromatic L-amino acid decarboxylase
PBS	phosphate-buffered saline
SON	supraoptic nucleus
TH	tyrosine hydroxylase
VMAT2	vesicular monoamine transporter 2
