# Supplementary material for: Partial Expression of Genes and Some Proteins Associated with the Dopaminergic Phenotype in Vasopressinergic Neurons of the Supraoptic Nucleus in Intact Mice
Source: Int J Mol Sci. 2026 Oct 6;27(19):8884. doi: 10.3390/ijms27198884 (PMC13665370; doi:10.3390/ijms27198884)
Supplement: Supplementary file 1 [file ijms-27-08884-s001.zip › ijms-4503048-supplementary.pdf]

Validation of primer pairs used for PCR.

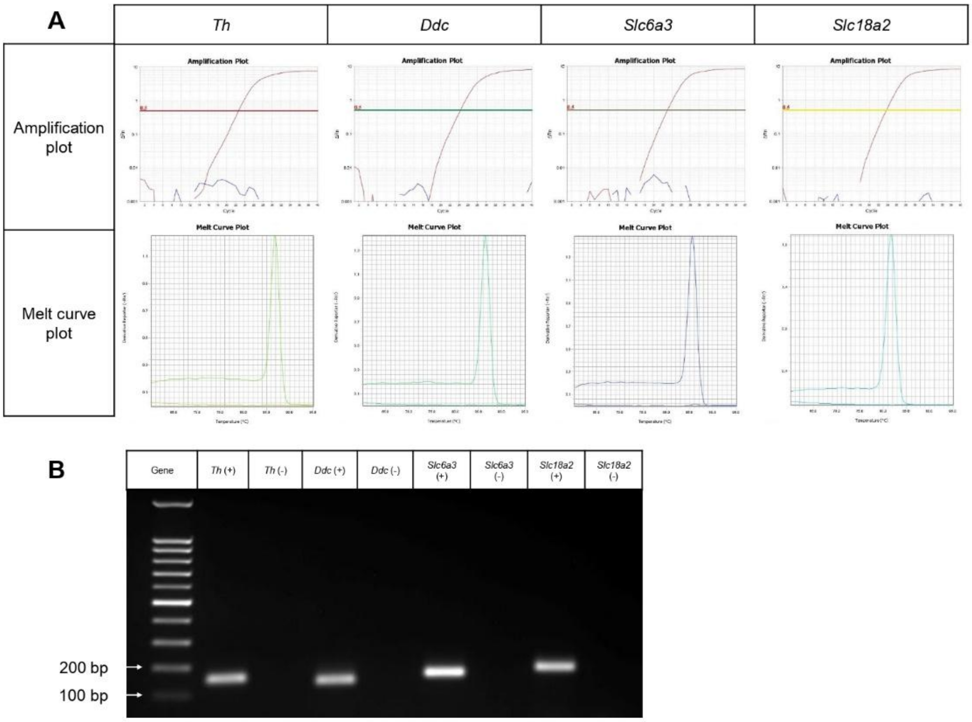

Figure S1. Validation of primer pairs used for PCR. (A) Amplification plots and melt curve plots for positive controls (tissue sample from the substantia nigra pars compacta containing cells expressing the gene of interest) and negative controls (no template—cDNA replaced with PCR-grade water). (B) Agarose gel electrophoresis of PCR products (amplification products separated on a 1.5% agarose gel stained with ethidium bromide; Lane 1: molecular weight marker (100–1500 bp DNA ladder); Lanes 2, 4, 6, 8: positive controls for *Th, Ddc, Slc6a3*, and *Slc18a2*; Lanes 3, 5, 7, 9: negative controls for *Th, Ddc, Slc6a3*, and *Slc18a2* (no template).
Abbreviations: bp, base pair; +, positive controls; -, negative controls.

Amplification plots in no-reverse-transcription controls

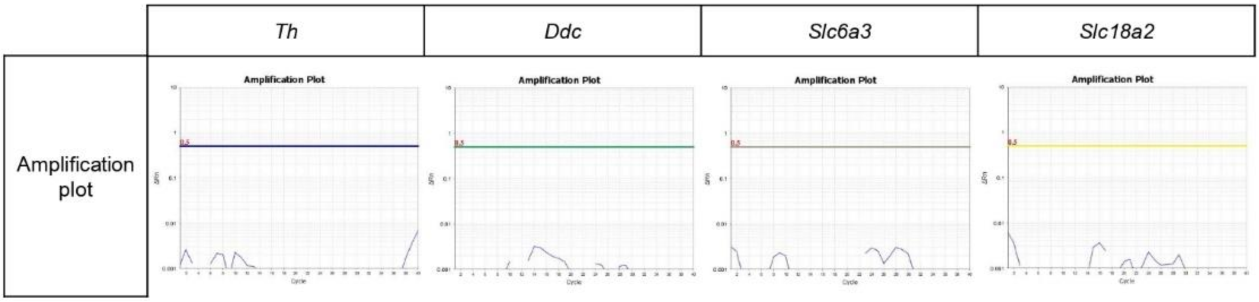

Figure S2. Amplification plots in no-reverse-transcription controls for the detection of *Th*, *Ddc*, *Slc6a3*, and *Slc18a2* following DNase I treatment of the samples.

Positive control for AADC and VMAT2 in substantia nigra

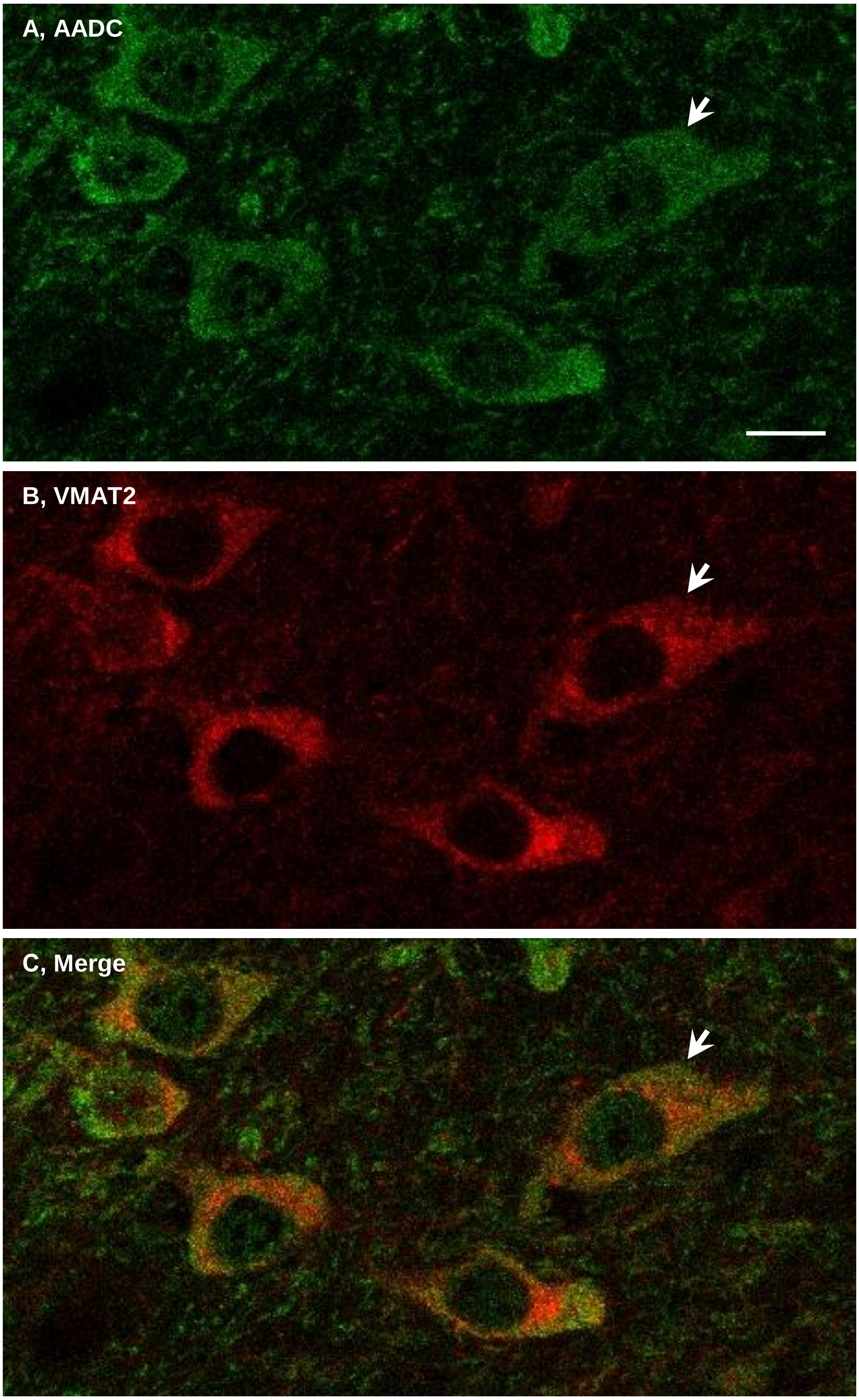

Figure S3. Positive control for immunostaining specificity for aromatic L-amino acid decarboxylase (AADC) (A, green, Alexa 488 antibodies) and vesicular monoamine transporter 2 (VMAT2) (B, red, Alexa 647 antibodies) and their colocaliztion (C, merge) in dopaminergic neurons of the substantia nigra of intact C57BL/6 mice. Confocal microscopy with a 63x objective. Scale bar: 10 µm.

Arrow, the representative dopaminergic neuron immunopositive for AADC and VMAT2.
